# Conductivity Classification of Non-Magnetic Tilting Metals by Eddy Current Sensors

**DOI:** 10.3390/s20092608

**Published:** 2020-05-03

**Authors:** Yue Du, Zhijie Zhang, Wuliang Yin, Shuang Zhu, Ziqi Chen, Hanyang Xu

**Affiliations:** 1 School of Instrument and Electronics, North University of China, Taiyuan 030051, China; duyue_nuc@163.com; 2 School of Electrical and Electronic Engineering, University of Manchester, Manchester M60 1QD, UK; Shuang.zhu@manchester.ac.uk (S.Z.); Ziqi.chen@manchester.ac.uk (Z.C.); hanyang.xu@postgrad.manchester.ac.uk (H.X.)

**Keywords:** non-magnetic metal, tilting, conductivity classification, eddy current sensors, photoelectric sensors, characteristic phase

## Abstract

Metallic waste classification benefits the environment, resource reuse and industrial economy. This paper provides a fast, non-contact and convenient method based on eddy current to classify metals. The characteristic phase to characterize different conductivity is introduced and extracted from mutual inductance in the form of amplitude and phase. This characteristic phase could offer great separation for non-tilting metals. Although it is hard to classify tilting metals by only using the characteristic phase, we propose the technique of phase compensation utilizing photoelectric sensors to obtain the rectified phase corresponding to the non-tilting situation. Finally, we construct a classification algorithm involving phase compensation. By conducting a test, a 95% classification rate is achieved.

## 1. Introduction

Eddy currents induced by time-varying magnetic fields have been widely applied to nondestructive defect detection, imaging, identification of metals and so forth [1,2,3]. To produce the eddy current, an alternating current excites a coil which generates the time-varying magnetic field by Ampère’s law [4]. According to Faraday’s law, an electric field around a closed loop is activated, and that drives electrons in metals to move, forming the eddy current [5]. There are several advantages of using eddy currents that greatly attract researchers: non-contact, non-destructive and flexible detection depth for inspection [6,7,8].

An eddy current is usually utilized to sort out metal from waste. For instance, magnetic deflecting force has been used to separate non-ferrous metals from electronic scrap [9,10]. The effects of particle size, particle shape and conductivity were also discussed. Nevertheless, the high-force eddy current separator in this paper had no ability to separate metals from each other. Aluminum is separated from a lithium-iron phosphate mixture by an eddy current in [11], which actually is far from the separation between different metals. Recently, eddy-current-based impedance spectroscopy was used to achieve fast classification of non-magnetic metals [12]. The authors introduced a method in which the real component of the secondary magnetic field with respect to the excitation magnetic field at 64 KHz and the imaginary component at 16 KHz were functioning as the *x*-axis and *y*-axis in a Cartesian coordinate system to give the best fitting function for each metal, by which, overall, 94.4% accuracy was realized. However, the discussion of tilting of metal samples (rotating along an axis in a Cartesian coordinate system) has not been involved, and merely copper, aluminum and brass have been classified, which are a fraction of metals used in industry.

In this paper, the characteristic phase of mutual inductance for a dynamic metallic sample is found. This characteristic phase offers satisfactory classification of five different samples under 40 KHz excitation frequency. Afterwards, a follow-up experiment on tilting metals is conducted. Although it is hard to classify tilting samples by only using characteristic phases, with the help of fitting a linear function of characteristic phase and tilting angle, we are able to utilize phase compensation to obtain rectified phase to achieve classification. The phase compensation is supported by using photoelectric sensors to obtain the voltage which is approximately proportional to the tilting angle. We finally construct a classification algorithm through which our test shows a 95% classification rate.

## 2. Theoretical Foundation

Dodd and Deeds [13] in 1968 gave the analytical solution of the model wherein a single coil is above a conductor, and calculated the induced voltage. Yin [14] further proposed the closed-form solution (1) to the mutual inductance between two coils [15].

(1) $$\Delta L\left(\omega \right)=K\int_{0}^{\infty }\frac{P^{2}\left(\alpha \right)}{\alpha^{6}}A\left(\alpha \right)\phi \left(\alpha \right)\;d\alpha$$

where

(2) $$\phi \left(\alpha \right)=\frac{\left(\alpha_{1}+\mu \alpha \right)\left(\alpha_{1}-\mu \alpha \right)-\left(\alpha_{1}+\mu \alpha \right)\left(\alpha_{1}-\mu \alpha \right)e^{2\alpha_{1}c}}{-\left(\alpha_{1}-\mu \alpha \right)\left(\alpha_{1}-\mu \alpha \right)+\left(\alpha_{1}+\mu \alpha \right)\left(\alpha_{1}+\mu \alpha \right)e^{2\alpha_{1}c}}$$

(3) $$\alpha_{1}=\sqrt{\alpha^{2}+j\omega \sigma \mu_{0}}$$

(4) $$K=\frac{\pi \mu_{0}N^{2}}{{\left(l_{1}-l_{2}\right)}^{2}{\left(r_{1}-r_{2}\right)}^{2}}$$

(5) $$P\left(\alpha \right)=\int_{\alpha r_{1}}^{\alpha r_{2}}xJ_{1}\left(x\right)dx$$

(6) $$A\left(\alpha \right)={\left(e^{-\alpha l_{1}}-e^{-\alpha l_{2}}\right)}^{2}$$

where α is the spatial frequency, ω is the excitation angular frequency, σ is the conductivity, $\mu_{0}$ is the vacuum permeability, *c* is the thickness of the sample, *N* is the number of turns of the coil, $l_{2}-l_{1}$ is the height of the eddy current sensor, $r_{1}$ is the radius of ferrite, $r_{2}$ is the radius of the eddy current sensor and $J_{1}$ is the bessel function of the first kind. As is shown in (1) and (3), the mutual inductance is the function of the conductivity, which is a component of the imaginary part. Thus, we conduct experiments trying to extract the phase information from mutual inductance to classify different conductivities.

## 3. Experimental Set-Up

**Figure 1:**
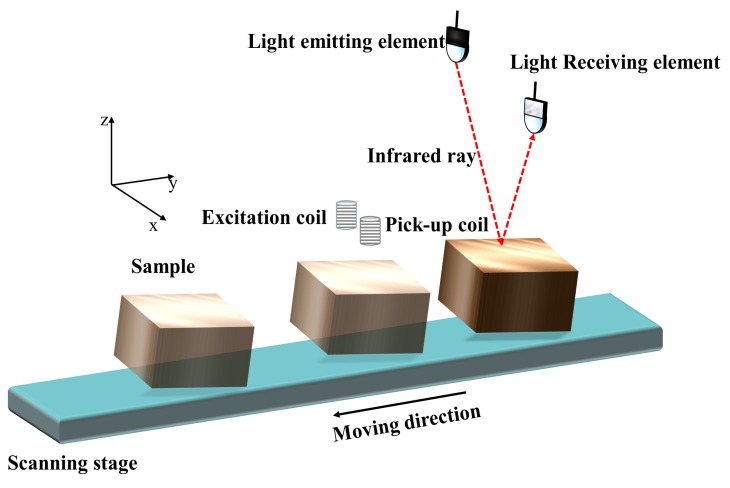
Setup model of the experiment.

**Figure 2:**
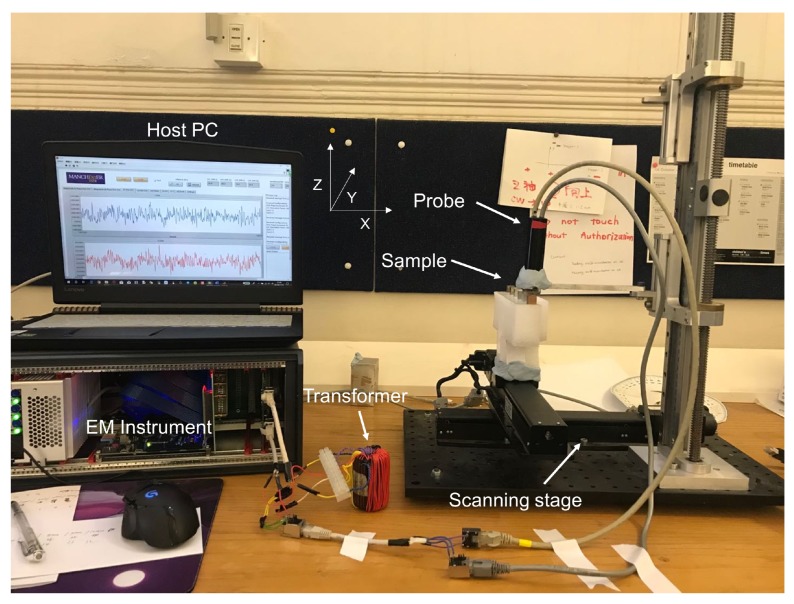
Photo of the entire separation system.

The types of metals used in the experiments were copper, aluminum, zinc, tin and titanium. Metal samples were all $1\times 1\times 1\;{\mathrm{cm}}^{3}$ cubes. We first conducted the experiment to classify non-tilting metals. We used the eddy current sensors in [16]. The setup of the experiment is shown in Figure 1 wherein the sample, rotating around *x*-axis, was moved along the *y*-axis under the eddy current sensors by a motion controller. The coils of eddy current sensors (excitation coil and pick-up coil) and elements of TCRT5000 photoelectric sensors (light emitting element and light receiving element) are located in the *x*–*z* plane. Figure 2 shows components of the entire separation system, which are eddy current sensors, a motion controller, an EM instrument in [17] and a computer. To acquire the mutual inductance, we use the calibration method introduced in [17], which is given by

(7) $$\Delta L=\frac{V_{sample}-V_{air}}{j\omega I}=\left(V_{sample}-V_{air}\right)\times \frac{M}{V_{transformer}}$$

where $V_{sample}$ is the induced voltage with a sample, $V_{air}$ is the induced voltage in free space, $V_{transformer}$ is the induced voltage in the transformer, *M* is the mutual inductance of the transformer, *I* is the excitation current and ω is the angular frequency of the excitation signal.

## 4. Non-Tilting Metal Classification

**Figure 3:**
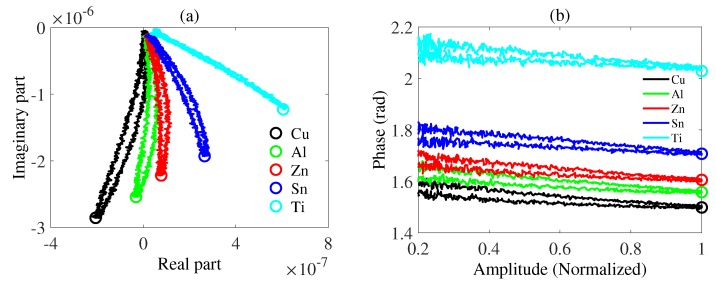
Measured mutual inductances when the excitation frequency is 40 KHz and the lift-off is 5 mm. (**a**) Mutual inductance in the form of a real part and an imaginary part. (**b**) Mutual inductance in the form of amplitude and phase. Trajectories are drawn, where black denotes copper, green denotes aluminum, red denotes zinc, blue denotes tin and cyan denotes titanium. The characteristic phase for each metal is marked by a circle with the same color as the corresponding trajectory.

We rewrite the measurements, which are composed of a real part and an imaginary part of mutual inductance, in the form of amplitude and phase.

(8) $$L_{r}+i*L_{i}=\left|L\right|e^{ip}$$

where $L_{i}$ is the imaginary part, $L_{r}$ is the real part, |L| is the amplitude and *p* is the phase. To compare different trajectories intuitively, we make the amplitude normalized. It is shown in Figure 3a that the mutual inductance values obtained in the form of real parts and imaginary parts for different samples have different trajectories, where the circle denotes the corresponding point of the characteristic phase which is mentioned in the next section. In detail, the trajectory would rotate counter-clockwise according to the magnitude of conductivity from copper to titanium. In other words, the smaller the conductivity, the larger the characteristic phase. Trajectories in the form of amplitude and phase are shown in Figure 3b for five metal samples. For instance, in fact, the left half of the mutual inductance trajectory for copper in Figure 3a is mapped to the bottom half of the trajectory in Figure 3b and the right half is mapped to the top half in Figure 3b. In Figure 3a, the route to get closer to the center of eddy current sensors provides the left half of the mutual inductance trajectory and that to get away from the center provides the right half. As is shown in Figure 3b, the trajectories are separated from each other and the order from bottom to top exactly follows the magnitude of conductivities of samples, as shown in Table 1. To characterize every different trajectory, we pay attention to the end point, which stays distinguishable. Thus, we choose the end point as the characteristic phase.

## 5. Tilting Metal Classification

**Figure 4:**
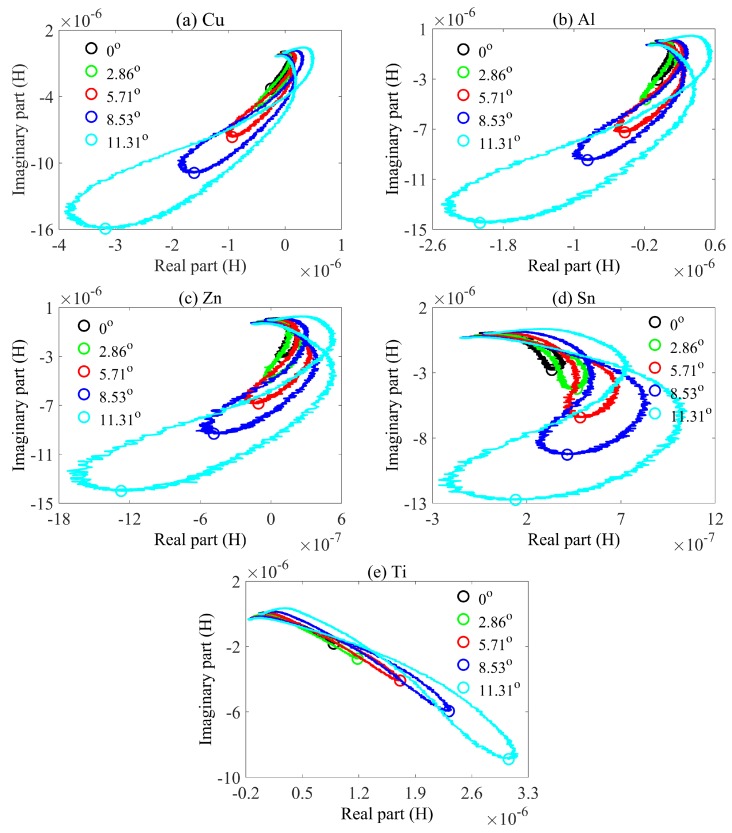
Measured mutual inductances in the form of real part and imaginary part for tilting samples. (**a**) Cu. (**b**) Al. (**c**) Zn. (**d**) Sn. (**e**) Ti. black line denotes $0^{\circ }$, green line denotes $2.86^{\circ }$, red line denotes $5.71^{\circ }$, blue line denotes $8.53^{\circ }$, cyan line denotes $11.31^{\circ }$. Circles are the points corresponding to characteristic phases.

The general case in metal classification is that the surfaces of wastes, where eddy current exists, are inclining—that is to say, not parallel to the sensors. The eddy current distribution in this situation becomes more complex. We first check the feasibility of keeping using characteristic phases to classify the oblique metals with the same set-up as shown in Figure 1. The mutual inductance trajectories for tilting samples are plotted in Figure 4 with 40 KHz excitation frequency. The lift-off in this experiment when the tilting angle is $0^{\circ }$ is 5 mm, which is kept constant for subsequent tilting samples.

**Figure 5:**
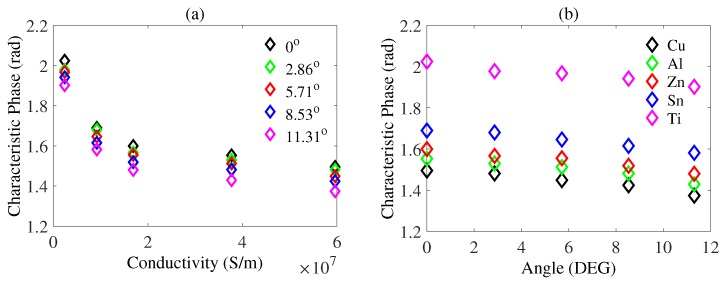
(**a**) Characteristic phase versus metals, where black diamond denotes $0^{\circ }$, green diamond denotes $2.86^{\circ }$, red diamond denotes $5.71^{\circ }$, blue diamond denotes $8.53^{\circ }$ and magenta diamond denotes $11.31^{\circ }$. (**b**) Characteristic phase versus angles where black diamond denotes copper, green diamond denotes aluminum, red diamond denotes zinc, blue diamond denotes tin and magenta diamond denotes titanium.

**Figure 6:**
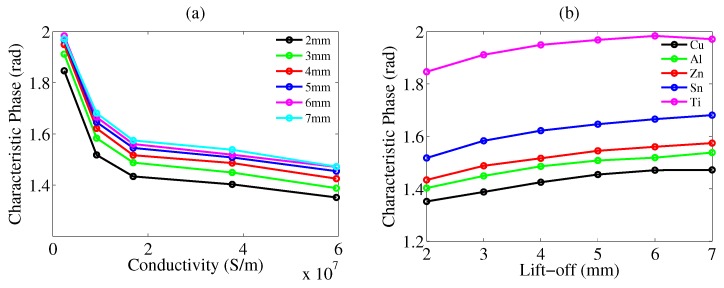
(**a**) Characteristic phase versus conductivity for different lift-off values. (**b**) Characteristic phase versus different lift-off values for different samples.

As is shown in Figure 5, as the tilting angle increases, the characteristic phase decreases for all the conductive samples. To explore whether the phase decrease was from angle or from lift-off, we performed an experiment in which lift-off was variable for non-tilting samples. As is shown in Figure 6, characteristic phase versus conductivity is plotted for lift-off varying from 2–7 mm.

**Figure 7:**
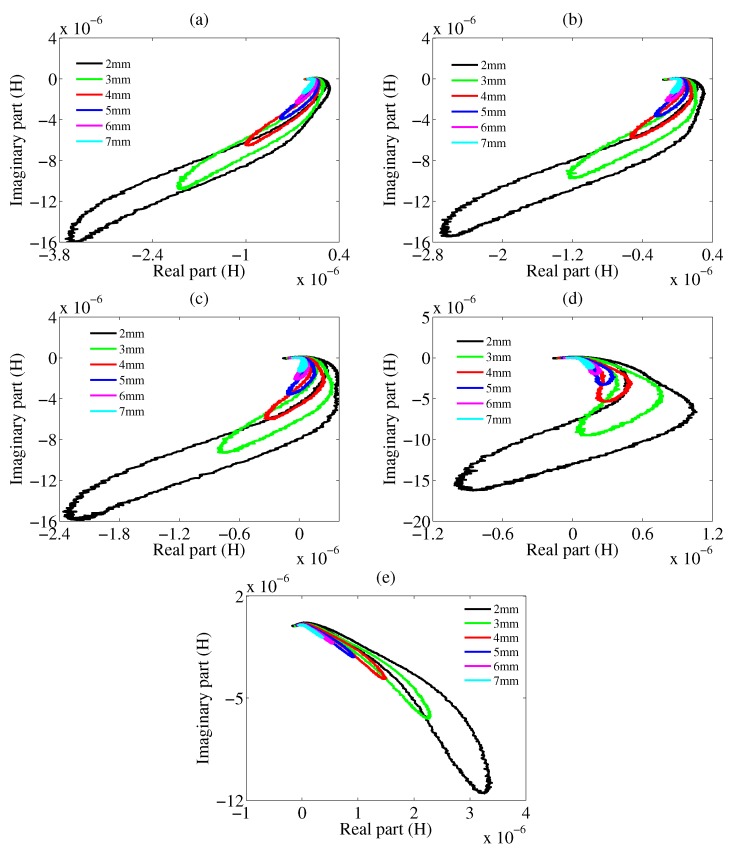
Measured mutual inductances in the form of the real part and the imaginary part for tilting samples with variable lift-off. (**a**) Cu. (**b**) Al. (**c**) Zn. (**d**) Sn. (**e**) Ti.

One conclusion can be drawn from Figure 6, which is that the characteristic phase rises up with lift-off. This conclusion matches up with the phase decrease when the angle is increasing, which actually corresponds to the lift-off decrease. Tilting causes the decrease of lift-off, thereby increasing the amplitude of receiving signal. This is why the lengths of trajectories in the same sub-figures of Figure 4 and Figure 7 are different. When the lift-off is larger or the tilting angle is smaller, the length of the trajectory becomes shorter. The physical reason is that a smaller lift-off results in a stronger eddy current because of the excitation electromagnetic field inversely proportional to $r^{3}$ where *r* is the distance between the sample and the excitation coil [18]. A larger eddy current would further enhance the secondary magnetic field, eventually increasing the mutual inductance. The equation for the relation between eddy current and secondary magnetic field is given by

(9) $$\oint B\cdot dl=\mu_{0}\;\int \int \;J\cdot dS$$

where J is the eddy current density and B is the secondary magnetic field produced by the eddy current. The relation between the secondary magnetic field and mutual inductance is given by

(10) $$L=\frac{\;\int \int \;B^{{}^{\prime }}\cdot dS}{I_{0}}$$

where $B^{{}^{\prime }}$ is the secondary magnetic field propagating to the pickup coil, $I_{0}$ is the excitation current and *L* is the mutual inductance.

Moreover, the characteristic phase range shown in Figure 5a (from the magenta diamond to the black diamond) for samples with different conductivities always has a part which coincides with others. This could become a problem where a tilting metallic sample may have the same characteristic phase as that of another sample. This issue exists for copper, aluminum, zinc, and tin but not for titanium. As is shown in Figure 5a, the smallest characteristic phase of titanium is larger than the rest. The method by which we address this problem is by using phase compensation, which will be comprehensively described in the next section.

## 6. Algorithm and Test

**Figure 8:**
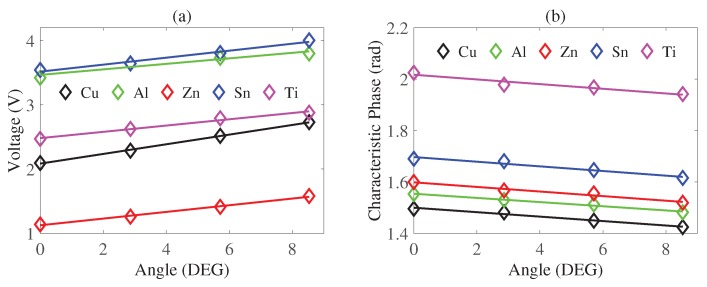
(**a**) Voltage versus angle where the black line denotes copper, the green line denotes aluminum, red line the denotes zinc, the blue line denotes tin and the magenta line denotes titanium. (**b**) Characteristic phase versus angles, where the black line denotes copper, the green line denotes aluminum, the red line denotes zinc, the blue line denotes tin and the magenta line denotes titanium.

**Figure 9:**
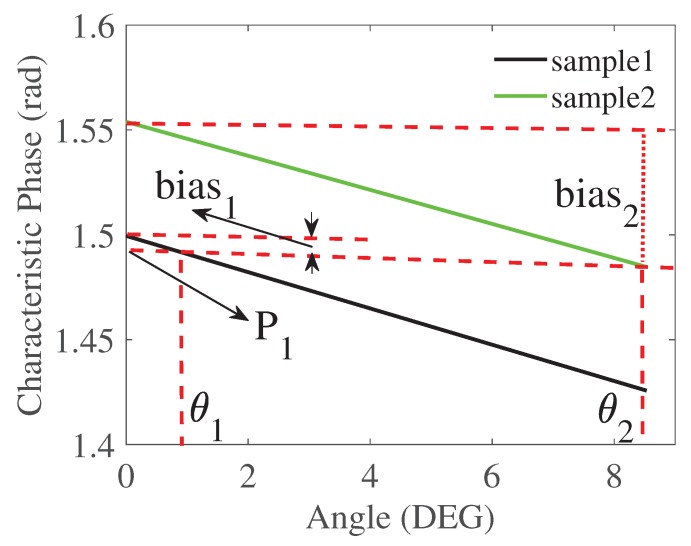
Schematic of the bias.

**Figure 10:**
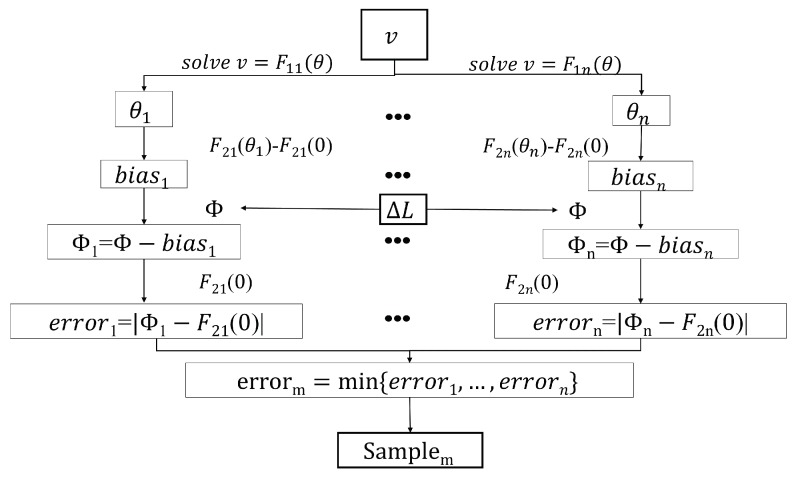
The flow diagram of the algorithm.

To identify the tilting angle, we propose utilizing photoelectric sensors because metal acts like a mirror for infrared rays which would be reflected back to the light-receiving element. When the metal is tilting, parts of the receiving signal would be reflected to free space instead of the light receiving element, which would decrease the irradiance on the phototransistor. However, the distance between the sample and the light receiving element is reduced by increasing the tilting angle, which would enhance the irradiance. Under small tilting angle, the net effect is the almost linearly increasing voltage measured by a voltmeter, as shown in Figure 8a. The voltage results from the photocurrent through a resistor connected to the phototransistor based on the amount of irradiance [19,20]. Therefore, so long as we find the voltage, the angle becomes known. It is shown in Figure 8a that with an angle smaller than $9^{\circ }$, the voltage $F_{1i}\left(\theta \right)$ could be taken as proportional to the angle, by which we are capable of finding the tilting angle by measuring the voltage of the resistor connected to the phototransistor. The method of least squares to obtain the fitting functions of voltage is given by

(11) $$F_{1i}\left(\theta \right)=k_{1i}\theta +b_{1i}$$

(12) $$\left[k_{1i},b_{1i}\right]=\underset{k_{1i},b_{1i}}{min}{∥F_{1i}-P_{i}∥}_{2}^{2}$$

where $F_{1i}$ denotes different fitting linear function of voltage for different samples, $k_{1i}$ denotes the slope and $b_{1i}$ denotes the y-intercept. As long as we find the tilting angle θ, we could post-process this phase with a bias to approach the characteristic phase in the non-tilting situation. Then we could achieve metal classification because the characteristic phases of different non-tilting samples are always differentiable. For example, as shown in Figure 9 (the green line and black line are chosen from Figure 8b, the sample2 under $\theta_{2}$ may have the same characteristic phase ($p_{1}$) as the sample1 under $\theta_{1}$. By resorting to the fitting functions of characteristic phase for the sample1 and sample2, we could find the $bias_{1}$ and $bias_{2}$. By subtracting the two different bias from $p_{1}$, the y-intercepts for two samples are obtained. Since the y-intercept corresponds to the characteristic phase when the tilting angle is $0^{\circ }$, we could always distinguish these y-intercepts as shown in Figure 8b. To find the fitting linear functions in Figure 8b for five samples, we use the method of least squares.

(13) $$F_{2i}\left(\theta \right)=k_{2i}\theta +b_{2i}$$

(14) $$\left[k_{2i},b_{2i}\right]=\underset{k_{2i},b_{2i}}{min}{∥F_{2i}-P_{i}∥}_{2}^{2}$$

where $F_{2i}$ denotes different fitting linear functions of the characteristic phases of different samples, $k_{2i}$ denotes the slope and $b_{2i}$ denotes the y-intercept. As long as we find the tilting angle θ, we could assign a bias to the detected characteristic phase. Thus, the final classifiable angle range is from $0^{\circ }$ to $9^{\circ }$. After obtaining those fitting functions, the bias is given by

(15) $$\delta_{i}=F_{2i}\left(\theta \right)-F_{2i}\left(0\right)$$

where θ is the detected angle and $F_{2i}\left(0\right)$ is the reference phase. The drawback here is that there may be several angles for one voltage, as shown in Figure 9. To resolve this issue, we need to form an algorithm, which is, step by step, as shown in Figure 10:

(1) Obtain the raw data of mutual inductance ΔL from eddy current sensors and voltage *v* from photoelectric sensors.
(2) Find the characteristic phase Φ and tilting angles through *v* for every possible sample; e.g., $\theta_{T}$ for tin and $\theta_{A}$ for aluminum.
(3) Find the bias for every tilting angle by (15), e.g., $\delta_{T}$ for $\theta_{T}$ and $\delta_{A}$ for $\theta_{A}$, and rectify the detected characteristic phase to get $\Phi_{k}$ of different metals; e.g., $\Phi_{1}=\Phi -\delta_{T}$ for tin and $\Phi_{2}=\Phi -\delta_{A}$ for aluminum.
(4) Calculate the errors $|F_{2k}\left(0\right)-\Phi_{k}|$ and find the metal type corresponding to the smallest error.

**Figure 11:**
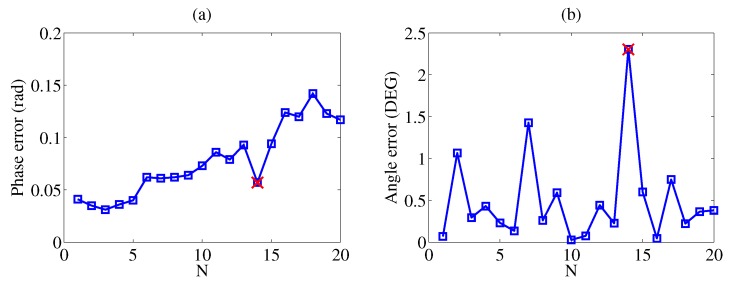
Test results (N is the order of testing) where red cross denotes the wrong test. (**a**) Phase error versus N. (**b**) Angle error versus N.

To test our classification algorithm, we did experiments twenty times to prepare the test data. As is shown in Figure 11, the largest angle error is near $1.5^{\circ }$ and the phase error is below 0.15 rad. The phase error and angle error are given by

(16) $$\Delta_{p}=min\{|F_{2k}\left(0\right)-\Phi_{k}\left|\right\}$$

(17) $$\Delta_{\theta }=\left|\theta -\theta^{{}^{\prime }}\right|$$

where $\Delta_{p}$ is the phase error, $\Delta_{\theta }$ is the angle error, θ is the actual angle and $\theta^{{}^{\prime }}$ is the angle calculated by the classification algorithm. If the output result is not the actual type of the sample we use, we count it as the wrong test; otherwise we count it as the right test. The classification rate is 95% but the angle error still needs improvement. The data about the wrong test are given in Table 2, from which it is obvious that the output result would be Al because the test error is smaller. However, the actual type of the sample we use in this test is Sn. The reason is that the fitting functions $F_{2i}$ for Al and Sn introduce different errors to the phase, as shown in Table 3. The train phase uses the data to obtain the fitting linear functions for characteristic phase, and the fitting error is the difference between the train phase and the fitting phase. The fitting error for Al is larger than that for Sn, which would affect the test error badly if the input phase of Sn were to some degree smaller than the train phase of Sn.

## 7. Conclusions

This paper achieves classification for five types of metals by characteristic phases of mutual inductance. This characteristic phase is obtained from the end point on the trajectory of mutual inductance in the form of amplitude and phase. By exploring trivial situation with non-tilting samples, we found that the characteristic phase is smaller for a sample with larger conductivity. Next, we turned our attention into tilting samples and found that trajectories have different lengths for different angles, and phase ranges of different samples intersect each other. By performing the experiment with variable lift-off, we concluded that the different size of the trajectory is related to the change of lift-off. To address the issue of phase overlap, we formed an algorithm to classify tilting metals. In this algorithm, we resort to the method of least squares to obtain two sets of linear functions, one of which is for the voltage measured by the voltmeter and the other is for the characteristic phase extracted from the mutual inductance. By inputting the voltage, we can find the tilting angle on which the bias is based. After acquiring the bias and the characteristic phase, the technique of phase compensation is applied. The last step is to compare the errors between the rectified phases and the reference phases and then output the type of metal according to the minimum error. This algorithm should be also applicable when more metals are included as long as they show similar linear relations for the voltage and the characteristic phase when the tilting angle is the variable. This algorithm can achieve classification under tilting angle not larger than $9^{\circ }$. The test results show a 95% classification rate. To improve the applied methodology, it will be possible to find another characteristic phase on the mutual inductance trajectory that could further decrease the fitting error; however, that is time-consuming. To make the classification intelligent, it is possible to resort to the machine learning or deep leaning methods since numerous phases are obtained in the dynamic experiments, which produce enough characteristics to train a classification model. In the future, we are interested in involving other variables, such as non-flat surfaces or irregular shapes, in our research to make the classification technique more generalized. Whether the characteristic phase and our algorithm are still applicable to the different alloys of metals is also attractive.

## Figures and Tables

**Table 1:** Conductivity (S/m) of metals.

Copper	Aluminum	Zinc	Tin	Titanium
$5.96\times 10^{7}$	$3.77\times 10^{7}$	$1.69\times 10^{7}$	$0.92\times 10^{7}$	$0.24\times 10^{7}$

**Table 2:** Data about the wrong test.

Input Voltage (V)	Angle (DEG)	Type	Input Phase (rad)	Fitting Phase (rad)	Test Error (rad)
3.81	5.40	Sn	1.61	1.70	0.09
3.81	8.01	Al	1.61	1.55	0.06

**Table 3:** Analysis about the wrong test.

Input Phase (rad)	Type	Train Phase (rad)	Fitting Phase (rad)	Fitting Error (rad)
1.61	Sn	1.65	1.70	0.05
1.61	Al	1.48	1.55	0.07

## References

[1] Yin L., Ye B., Rodriguez S., Leiva R., Meng X., Akid R., Yin W., Lu M. (2018). Detection of corrosion pits based on an analytically optimised eddy current sensor. Insight-Non-Destr. Test. Cond. Monit..

[2] Wu J., Zhu J., Xia H., Liu C., Huang X., Tian G.Y. (2019). DC-biased magnetization based eddy current thermography for subsurface defect detection. IEEE Trans. Ind. Inform..

[3] Xu H., Lu M., Avila J., Zhao Q., Zhou F., Meng X., Yin W. (2019). Imaging a weld cross-section using a novel frequency feature in multi-frequency eddy current testing. Insight-Non-Destr. Test. Cond. Monit..

[4] Karakoc K., Suleman A., Park E.J. (2016). Analytical modeling of eddy current brakes with the application of time varying magnetic fields. Appl. Math. Model..

[5] Nagel J.R. (2017). Induced eddy currents in simple conductive geometries: mathematical formalism describes the excitation of electrical eddy currents in a time-varying magnetic field. IEEE Antennas Propag. Mag..

[6] Xie L., Gao B., Tian G., Tan J., Feng B., Yin Y. (2019). Coupling pulse eddy current sensor for deeper defects NDT. Sens. Actuators Phys..

[7] Bouloudenine A., Feliachi M., Latreche M.E.H. (2017). Development of circular arrayed eddy current sensor for detecting fibers orientation and in-plane fiber waviness in unidirectional CFRP. NDT Int..

[8] Yu Y., Zhang D., Lai C., Tian G. (2017). Quantitative approach for thickness and conductivity measurement of monolayer coating by dual-frequency eddy current technique. IEEE Trans. Instrum. Meas..

[9] Smith Y.R., Nagel J.R., Rajamani R.K. (2019). Eddy current separation for recovery of non-ferrous metallic particles: A comprehensive review. Miner. Eng..

[10] Zhang S., Forssberg E., Arvidson B., Moss W. (1998). Aluminum recovery from electronic scrap by High-Force® eddy-current separators. Resour. Conserv. Recycl..

[11] Bi H., Zhu H., Zu L., Gao Y., Gao S., Wu Z. (2019). Eddy current separation for recovering aluminium and lithium-iron phosphate components of spent lithium-iron phosphate batteries. Waste Manag. Res..

[12] O’Toole M.D., Karimian N., Peyton A.J. (2017). Classification of nonferrous metals using magnetic induction spectroscopy. IEEE Trans. Ind. Inform..

[13] Dodd C., Deeds W. (1968). Analytical solutions to eddy-current probe-coil problems. J. Appl. Phys..

[14] Yin W., Binns R., Dickinson S.J., Davis C., Peyton A.J. (2007). Analysis of the liftoff effect of phase spectra for eddy current sensors. IEEE Trans. Instrum. Meas..

[15] Yin W., Peyton A. (2007). Thickness measurement of non-magnetic plates using multi-frequency eddy current sensors. NDT Int..

[16] Xu H., Avila J.R.S., Wu F., Roy M.J., Xie Y., Zhou F., Peyton A., Yin W. (2018). Imaging x70 weld cross-section using electromagnetic testing. NDT Int..

[17] Chen Z., Salas-Avlia J.R., Tao Y., Yin W., Zhao Q., Zhang Z. (2020). A novel hybrid serial/parallel multi-frequency measurement method for impedance analysis in eddy current testing. Rev. Sci. Instrum..

[18] Balanis C.A. (2016). Antenna Theory: Analysis and Design.

[19] Tavernier F., Steyaert M. (2011). High-Speed Optical Receivers with Integrated Photodiode in Nanoscale CMOS.

[20] Williams J. (2017). The Crystal Triode: The Transistor. The Electronics Revolution.

